# Overcoming Barriers to the Market Access of Biosimilars in the European Union: The Case of Biosimilar Monoclonal Antibodies

**DOI:** 10.3389/fphar.2016.00193

**Published:** 2016-06-29

**Authors:** Evelien Moorkens, Clara Jonker-Exler, Isabelle Huys, Paul Declerck, Steven Simoens, Arnold G. Vulto

**Affiliations:** ^1^Department of Pharmaceutical and Pharmacological Sciences, University of LeuvenLeuven, Belgium; ^2^Hospital Pharmacy, The Erasmus University Medical CenterRotterdam, Netherlands

**Keywords:** biosimilar, monoclonal antibody, market access, European Union, literature review

## Abstract

**Background:** In 2014, six of the top ten blockbuster medicines were monoclonal antibodies. This multibillion-dollar market with expiring patents is the main driver for the development of biosimilar mAbs. With the ever-increasing cost of healthcare and the economic pressure to reduce or sustain healthcare expenses, biosimilars could be instrumental in reducing costs for medication and increasing patient access to treatment.

**Objectives:** The aim of this study is to identify and describe the barriers to market access of biosimilar mAbs in the European Union and to analyze how these barriers could be overcome.

**Methods:** A narrative literature review was carried out using the databases PubMed, Embase, and EconLit. Studies were published in English or Dutch. Additionally, the reference list of the articles was checked for relevant studies. Articles and conference papers known to the authors were included as well. Articles were also identified by searching on the website of the Generics and Biosimilars Initiative (GaBI) journal.

**Results:** Six barriers were identified based on available literature: The manufacturing process, the regulatory process, intellectual property rights, lack of incentive, the impossibility of substitution, and the innovator's reach. These six barriers are presented as a possible framework to study the market access of biosimilar mAbs. Based on the literature search, recommendations can be made to overcome these barriers: (i) invest initially in advanced production processes with the help of single-use technology, experience or outsourcing (ii) gain experience with the regulatory process and establish alignment between stakeholders (iii) limit patent litigation, eliminate evergreening benefits, build out further the unitary patent and unified patent litigation system within the EU (iv) create demand-side policies, disseminate objective information (v) change attitude toward biosimilar switching/substitution, starting with physician, and patient education (vi) differentiate the biosimilar by service offerings, use an appropriate comparator in cost-effectiveness analyses.

**Conclusions:** Barriers to the market access of biosimilar mAbs could be reduced when more transparency and communication/education is used in all steps toward market access in order to increase the trust in biosimilar mAbs by all stakeholders. Only then biosimilar mAbs will be able to fully capture their cost saving potential.

## Introduction

Biotechnology has been widely adopted by large pharmaceutical companies in the development of new medicines. Medicines produced using biotechnology, biological medicines or biologics, represent a growing share of all medicines worldwide. In 2012, they accounted for 18% of the global market. In 2007, this was 15%, and in 2002 this was only 11% (Rickwood et al., 2013). Monoclonal antibodies (mAbs) are a great contributor to this growth. Today they represent a multibillion-dollar industry of medicines used mainly in treatment of autoimmune diseases and cancer. In 2014, eight of the top ten blockbuster medicines in Europe were biological medicines, six of these were mAbs (Dolan, 2015). Due to the complex and costly manufacturing process and often unique therapeutic value, biological medicines are more expensive than small molecule chemical medicines. The high prices and the success of biological medicines put pressure on healthcare expenses, and this cost pressure may lead to a decrease in patient access to medicines (McCamish and Woollett, 2012; Rickwood and Di Biase, 2013).

Following expiry of patent and other exclusivity rights, the market may open to non-innovator versions of biological medicines, so-called biosimilar medicines or biosimilars, which tend to be less expensive due to a lower research and development (R&D) cost and possible impact of competition. In the European Union, the European Medicines Agency (EMA) has established a regulatory framework for registration of biosimilars since 2005. The EMA defines a biosimilar as follows: “*A biosimilar is a biological medicinal product that contains a version of the active substance of an already authorized original biological medicinal product (reference medicinal product) in the European Economic Area (EEA). Similarity to the reference medicinal product in terms of quality characteristics, biological activity, safety and efficacy based on a comprehensive comparability exercise needs to be established.”* (EMA, 2014) Later, product specific biosimilar guidelines were developed, e.g., for products containing monoclonal antibodies (EMA, 2012a). In September 2013, the first biosimilar mAb for infliximab developed by Celltrion was approved by the EMA as Inflectra® (by Hospira) and Remsima® (by Celltrion). These products could not enter the greater part of the European market until February 2015 due to prolongation of market exclusivity of the innovator medicine Remicade®. In 2014 global sales of the originator product Remicade® accounted for $9.8 billion (2014 Sales of Recombinant Therapeutic, Antibodies and Proteins, 2015). Even a limited price discount of 30% would create a substantial cost-saving per treatment.

With the ever-increasing cost of healthcare and the economic pressure to reduce or sustain healthcare expense, biosimilars could be instrumental in reducing cost for medication and increasing patient access to treatment (McCamish and Woollett, 2012; Rickwood and Di Biase, 2013). Especially the cost for cancer treatment is becoming unaffordable, even for wealthy countries, due to higher prevalence of cancer and more expensive biological and targeted therapies (Cornes, 2012). This results in a huge market potential for biosimilars.

The advancement in technology makes biosimilars possible and the pressure on healthcare budgets makes them desirable. Therefore, both a technological push and a market pull mechanism are facilitating biosimilar mAb development. In comparison to earlier introduced biosimilars, e.g., filgrastim biosimilars, biosimilar mAbs are more complex in structure and mode of action. In addition, competition from innovative products is fierce, and the regulatory requirements for biosimilar mAbs are more complex than for smaller biosimilars (Declerck, 2013; Mellstedt, 2013). The huge market potential nevertheless resulted in many pharma companies investing in biosimilar mAb development.

Although multiple patents of mAbs are expired (e.g., rituximab, trastuzumab; GaBI, 2015), so far only one biosimilar mAb received marketing authorization, i.e., a biosimilar of infliximab (European Medicines Agency, 2016a). Biosimilar etanercept was approved early 2016 and is regarded by many as a mAb. However, etanercept in a strict sense is not a mAb, but a TNF receptor fusion protein. It was assessed by EMA according to the same principles as mAbs (European Medicines Agency, 2015a). We expect the same uptake barriers for biosimilar etanercept. Furthermore, earlier biosimilars have seen slow uptake in European markets (GaBI, 2012; Farfan-Portet et al., 2014). With the slow emergence and uptake of biosimilar mAbs, we perceive that market access of biosimilar mAbs is hindered by several barriers.

The aim of this study is to (a) identify the barriers to market access of biosimilar mAbs in the European Union (EU), and (b) analyze how these barriers could be overcome.

This manuscript is based on the MBA thesis of one of the authors, Clara Jonker-Exler (Jonker-Exler, 2014). To the best of the authors' knowledge, this is the first study that (a) reviews the literature on market access of a significant class of new medicines, i.e., biosimilar mAbs, (b) uses a framework to identify and discuss barriers to market access of biosimilar mAbs, and (c) proposes clear recommendations to reduce or remove such barriers.

## Methods

This study is based on a narrative literature review. Relevant studies were identified by searching PubMed, Embase and EconLit up to November 2015. The following search terms were used: “biosimilar monoclonal antibody,” “biosimilar market entry.” Studies could be published in English or Dutch. Additionally, the reference list of the articles was checked for other relevant studies. Articles known to the authors were included as well and conference papers were scrutinized for relevant information. An additional source was the website of the Generics and Biosimilars Initiative (GaBI) journal, GaBI Online.

Published literature was analyzed while the biosimilar mAb market is developing faster than studies can be carried out and published. The barriers may resolve or change over time and there will possibly be differences between different types of biosimilar mAbs. Even though only one biosimilar mAb is currently on the market, patents expired for some of the highest sold biologicals (rituximab, trastuzumab) or will expire in the next few years (Table 1), allowing more biosimilar mAbs to enter the market. Currently (March 2016), four dossiers for biosimilar mAbs [infliximab (one), rituximab (one) and adalimumab (two)] are under review at the EMA (European Medicines Agency, 2016c).

**Table 1:** **Exclusivity expiration dates monoclonal antibodies in the European Union (EU) (GaBI, 2015; European Medicines Agency, 2015c)**.

**INN**	**Reference product**	**Exclusivity expiration EU**	**Biosimilar**	**Year of EMA approval**
Infliximab	Remicade	2015		1999
			Inflectra	2013
			Remsima	2013
Adalimumab	Humira	2018	/	2003
Etanercept^*^	Enbrel	2015		2000
			Benepali	2016
Rituximab	Rituxan	2013	/	1998
Trastuzumab	Herceptin	2014	/	2000
Bevacizumab	Avastin	2022	/	2005
Cetuximab	Erbitux	2014	/	2004
Ranibizumab	Lucentis	2022	/	2007

* *Not a real mAb*.

## Results and discussion

### Barriers to market access

Since literature on the market access of biosimilar mAbs is not abundant, this paper describes the market access barriers for biosimilars in general in case specific information on biosimilar mAbs was not available.

With a view to undertaking a structured analysis of barriers to biosimilar market access, this paper draws on the framework proposed by Aaron (Ronny) Gal and the hurdles that he identified (Gal, 2014; Gal et al., 2015). Gal's framework was adapted based on the available literature to develop our own framework that allows to analyze barriers to market access of biosimilar mAbs. Six barriers were identified based on available literature and hurdles described by Aaron (Ronny) Gal (Gal, 2014; Gal et al., 2015): The manufacturing process, the regulatory process, intellectual property rights, lack of incentive, the impossibility of substitution, and the innovator's reach. (Table 2) These six barriers are presented as a possible framework to study the market access of biosimilar mAbs.

**Table 2:** **Barriers to market access of biosimilar monoclonal antibodies in the European Union**.

Manufacturing process	• Expensive • Complex
Regulatory process	• Uneven contribution and acceptation by stakeholders
Intellectual property rights	• Innovator patents • Prolongation of exclusivity rights • Patent disputes
Lack of incentive	• Difficult to differentiate • Limited price discounts • Limited knowledge and acceptance • Burden of change
Impossibility of substitution	• No interchangeability • Little to no policies in favor of switching and substitution
Innovator's reach	• Strong ties with physicians and patients • Competitive rebates

#### An expensive and complex manufacturing process

The barrier of scale and experience is high in the biotech pharma industry, mainly because of high investment requirements for manufacturing processes and learning curve effects (Simoens, 2009).

Not only the cost, but also the complexity of the manufacturing process hinders biosimilar mAb development. Biosimilar developing companies have no access to the original biological expression system that was used for the innovative reference product and therefore need to take a reverse engineering approach (Mellstedt, 2013). Thus, full knowledge of the production process of the innovator medicine is not available as it is mainly protected by trade secret. Contrary to highly predictable chemical processes, the biological production process is more difficult to control (Lepage-Nefkens et al., 2013), and quality attributes of the product are highly influenced by changes in the production process, e.g., different expression system, growth conditions,… (Declerck, 2013). This complicates the reverse engineering of biological medicines, and therefore the production of a highly similar biological medicine.

Once the reverse engineering of the biosimilar mAb is finished, and the development and testing is successful, the company will need to upscale production and possibly open new facilities to meet market demand. Every change in production, including upscaling, may affect one or more quality attributes of the product, and managing the manufacturing process will be a continuous challenge for the biosimilar developer. Hence, access to manufacturing technologies may be restricted to those companies who have been investing in biologic manufacturing platforms throughout the years (Calo-Fernández and Martínez-Hurtado, 2012).

#### A regulatory process with uneven contribution and acceptation by stakeholders

The EMA guideline for biosimilar mAbs was approved in 2012 (EMA, 2012a). Until that time, biosimilar developers were unsure about the final regulatory requirements for biosimilar mAbs. In 2009, EMA started with providing individual protocol assistance and scientific consultations to companies to avoid slowdown of the development of biosimilar mAbs (European Medicines Agency, 2015b). Even with the published guideline, the evaluation is very much case-by-case and the developer has the opportunity to propose novel study techniques (Schellekens and Moors, 2010; EMA, 2012a).

During the public consultation period of the EMA guideline on biosimilar mAbs, industry and regulatory representatives made comments, while the medical profession did not take this opportunity to be involved in designing the guidelines (Ebbers et al., 2012a). This uneven contribution to the guidelines by different stakeholders has led to uneven commitments of medical professionals, patients and industry to the outcome of the guideline. This may have contributed to concerns raised by medical professionals and patients/patient organizations, subsequently hindering uptake of biosimilars in the market (Aapro, 2011).

#### Innovator patents, prolongation of exclusivity rights, and patent disputes

The earliest market entry date for a biosimilar is the latest expiration date of relevant patents as well as data and market exclusivity rights, but in most cases patents are the determinant blocking market entry (Rader, 2013). Patent protection is a legal restraint on new market entry of biosimilars, but patent protection and market exclusivity are tools for innovator biologicals to recover the R&D expense.

While the patent for a product, which claims the specific amino acid sequence of the medicine, blocks market entry of biosimilars, innovator patents on production processes and mAb applications complicate the development of biosimilars. Patents on manufacturing processes can be used to prevent manufacturers of biosimilars from using the same production processes as innovators. On the other hand, biosimilar developers will never know the details of the production process, since these are kept as a trade secret. Choosing or adapting different production processes may lead to differences in the end product, which then need to be shown as not having an effect on efficacy and safety in patients. This adds to the burden and cost of manufacturing process design of biosimilars and validation after manufacturing.

The first approved biosimilar mAb, an infliximab biosimilar, could not directly enter the greater part of the European market due to an extension of market exclusivity of the innovator medicine Remicade® of 6 months, granted in return of filing an extra indication for pediatric use (GaBI, 2013). The possibility of prolongation of exclusivity rights, as a reward for e.g., licensing pediatric indications, makes the date for market entry of the biosimilar more uncertain. In addition, patents are territorial in scope, therefore patent expiration dates may differ across countries, which further complicates market entry.

The first company to launch a biosimilar will likely need to resolve patent disputes and this could explain why some companies have halted their trials (Rader, 2013; Malkin, 2015). This leads to a complicated trade-off between postponement of the launch date, investing high budgets in new production processes and subsequent validation, or risking the cost and possible loss of a patent dispute.

With the slow uptake of existing biosimilars and the difficulties in developing biosimilar mAbs, the abrupt drop in sales or patent cliff that innovators face is much less sharp and acute for biologics than in the case of generic small molecules (Calo-Fernández and Martínez-Hurtado, 2012). Nevertheless, with the high revenues of biologics threatened by biosimilar competition, the innovator companies are expected to fight off and delay this competition.

#### Difficulties to differentiate a biosimilar, low price discounts, limited knowledge and acceptance, and the burden of change lead to a lack of incentive

After marketing authorization is granted, the uptake of biosimilars is influenced by incentives for healthcare payers, physicians, pharmacists and patients to promote, prescribe, dispense, and use these biosimilars.

The possible strategy of differentiation leads to the importance of providing a biosimilar product with a greater perceived value than the originator biological product. Where innovative biologics are highly branded it is more difficult to differentiate biosimilars and use this to incentivize buyers.

With the high cost of development, the biosimilar can only be introduced on the market with a limited discount compared to the originator. They are usually priced at a discount of only 10–35% (Farfan-Portet et al., 2014). Absolute cost savings could still be substantial because of the high price and high volume of the reference medicines (Declerck and Simoens, 2012).

Physicians are hesitant to accept biosimilars as equal to the reference product (Aapro, 2011) and they generally do not directly benefit from the lower cost. There is a need for an incentive to facilitate biosimilar introduction and transition from prescribing the familiar and trusted reference product, to prescribing a biosimilar mAb with its inherent uncertainties, and to compensate for the effort it will take to explain to their patients the switch to a cheaper alternative. Patients will follow the advice of their physician and patient associations, and are influenced by reports in general media.

Physicians are mainly concerned about patient safety and need to choose the right treatment, based on available information (Schellekens, 2009). The overall slow uptake of currently available biosimilars in Europe is mainly ascribed to low physician knowledge and acceptance of the concept of biosimilarity (Aapro, 2011; European Commission, 2013; Rickwood and Di Biase, 2013).

#### No interchangeability, and little to no policies in favor of switching and substitution

Another barrier biosimilar mAbs face is the impossibility of substitution in most European countries. Substitution is the act of replacing the innovator medicine with the biosimilar, or one biosimilar for the other, at the pharmacy level, without the previous consent of the prescribing physician (Boone et al., 2013). Substitution is the reason generic medicines can gain market share rapidly.

In Europe, the national authorities are responsible for the legislation on substitution. So far, few countries, e.g., France and Germany, have explicitly allowed a restricted form of biosimilar substitution (Drozd et al., 2014; GaBI, 2014) and therefore market share in Europe will need to be gained by costly marketing or high discounts.

The decision on interchangeability lies as well with the national authority of the EU member states. The evaluation by the EMA does not include a statement on interchangeability of biosimilars on an individual level (EMA, 2012b). Interchangeability of the biosimilar is a prerequisite for substitution. It could be argued that if a biosimilar is approved in Europe, it is deemed interchangeable with its reference product on a population level (Ebbers et al., 2012b). Even though this is applicable to treatment-naïve patients, uncertainties still exist about switching for patients during their treatment.

Switching is prescribing or dispensing a biosimilar to a patient who was previously using the innovator medicine, or vice versa. To enable track and trace, repeated switching and substitution without consent of the prescribing physician are not advised (Weise et al., 2012). A company can only live up to its post-authorization safety study requirements if accurate track and trace is guaranteed, so an adverse event can be traced to the exact product causing it (Vermeer et al., 2015). It should be noted that this is further complicated by the delay of certain adverse events such as immunogenicity. If more biosimilars of the same molecule enter the market, the issue is further complicated, as a biosimilar is similar to a reference product, and similarity to another biosimilar has not been studied.

Without the possibility of substitution, biosimilars are offered as a choice for new patients or as a one-time switch for stable patients only. This market segmentation makes the size of the potential market for the biosimilar a fraction of the total market of the reference product, especially for those products used in long-term treatments (Rickwood and Di Biase, 2013).

#### Strong ties of the innovator company with physicians and patients, and offering of competitive non-transparent rebates

As mentioned before, the innovator company is likely to undertake steps to protect its market share against biosimilar mAb competition (Morisot et al., 2013), and has a better position to offer a differentiated product. The innovator companies protect their market share not only by patent strategies but also through strong ties with physicians and patients.

The innovator companies often have a long lasting relationship with the physicians, sponsoring clinical research or offering practical support. Also patient associations often have strong ties with innovator companies that sponsor their meetings and offer educational materials.

For many European hospitals, the procurement of medications is done by a group of hospitals through extensive negotiation with the pharmaceutical industry (Lepage-Nefkens et al., 2013). High rebates or interesting research sponsoring can be given when procurement of medication is concentrated to a few companies. This can make it difficult for a biosimilar developer who might not offer as complete a package and is not able to give the rebate. The extent of the rebates, and other favorable agreements with hospitals, is often unknown to the third party payer (Lepage-Nefkens et al., 2013). For mAbs that are mainly used inside the hospital, it may therefore, depending on the funding model of the hospital, be profitable for the hospital to procure the innovator medicine, even if the biosimilar has a lower list price (and subsequent lower reimbursement rates). High-cost cancer medication like mAbs, are often not discounted, because many have no therapeutic alternative and they are not likely to be continued for outpatient use (Vogler et al., 2013). This can change when biosimilar mAbs enter the market offering a lower cost therapeutic alternative. The innovator company will likely lower the price of the innovator medicine and therefore biosimilar mAbs cannot rely on price competition alone but will need to offer a differential advantage through marketing and service offerings (Bocquet et al., 2014).

### Overcoming the barriers

Based on the literature review, the following recommendations are proposed to overcome barriers to the market access of biosimilar mAbs (Table 3).

**Table 3:** **Recommendations to overcome barriers to market access of biosimilar monoclonal antibodies in the European Union**.

**Barrier**	**Recommendation**
Manufacturing process	• Invest initially in advanced production processes with the help of single-use technology, buy-in of experience or outsourcing
Regulatory process	• Gain experience with the regulatory process and establish alignment between stakeholders
Intellectual property rights	• Limit patent litigation • Eliminate evergreening benefits • Build out further the unitary patent and unified patent litigation system within the European Union
Lack of incentive	• Create demand-side policies • Disseminate objective information
Impossibility of substitution	• Change attitude toward biosimilar switching/substitution, starting with physician and patient education
Innovator's reach	• Differentiate the biosimilar by service offerings • Use an appropriate comparator in cost-effectiveness analyses

#### Invest initially in advanced production processes with the help of single-use technology, buy-in of experience, or outsourcing

Technological advances have led to more efficient cell lines that produce an increased amount of antibody while using more standardized, less expensive media and thus creating a higher yield at a lower cost (Calo-Fernández and Martínez-Hurtado, 2012; Gal et al., 2015). The innovator company is bound by the validated original processes, while the biosimilar developer can use modern techniques from the start.

The availability of single-use (i.e., disposable) technology in the development stage of biosimilars may lower the cost involved in the early development stage (Whitford, 2012).

Further development cost and time savings can be realized through buy-in of manufacturing experience and outsourcing. Initial investment in a technically advanced production process will keep production costs low in the future and lead to a better competitive position when the biosimilar market becomes generic like with competition based on price (Rader, 2013).

With the ongoing technological advances in analysis and manufacturing of biosimilars, this barrier will decrease over time.

#### Gain experience with the regulatory process and establish alignment between stakeholders

The EMA guidelines evolved as a compromise between the different industrial parties, innovator vs. biosimilar, and the regulatory body. Regulatory authorities need to ensure patient safety and encourage both competition and innovation in the biopharmaceutical industry (Blackstone and Fuhr, 2013; European Medicines Agency, 2016b). The EMA guidelines are supportive of biosimilar development and it is up to the company developing the biosimilar to use it to the greatest advantage and to reduce the need for large and expensive clinical trials.

The historical market approval of biosimilars shows a case-by-case approach and approval of the marketing authorization application based on the available test data and the intellectual judgment of the EMA (Schellekens and Moors, 2010). Profound knowledge of and experience with the regulatory requirements, in combination with EMA Scientific advice/Protocol assistance, will help a company to adjust the biosimilar mAb development process to these requirements and overcome this barrier for the EMA regulatory pathway.

Greater alignment between the medical community and the regulators can lead to greater trust in the regulatory process (Ebbers et al., 2012a).

#### Limit patent litigation, eliminate evergreening benefits, build out further the unitary patent and unified patent litigation system within the EU

Innovator companies have protected their mAbs with a myriad of patents and will likely challenge infringement of these patents by biosimilar developers. The outcome of these patent disputes will provide jurisprudence for future cases.

Dylst et al. provide a list of recommendations to enhance market access of generic medicines (Dylst et al., 2012). Of these recommendations the following are equally applicable to biosimilars: (a) Grant patents only for truly innovative medicines, thereby eliminating evergreening benefits (i.e., follow-on patents on existing products for non-significant therapeutic improvements), (b) Create a unitary European Union patent, and (c) Unified patent litigation within the European Union. Plans for a unitary patent and unified patent litigation are already made (EPO, 2016). Several EU member states signed the Agreement on a Unified Patent Court (EU, 2013).

#### Create demand-side policies, disseminate objective information

Positive experience with available biosimilars (Vulto and Crow, 2012) is increasing confidence with payers and physicians and will positively influence uptake. Not all physicians are familiar with the concept of biosimilars, neither are they confident about their safety and efficacy, but awareness has increased over the years (Noaiseh and Moreland, 2013). Providing objective information about the characteristics of biosimilars is key in increasing physician acceptance (Lepage-Nefkens et al., 2013). Improved communication to physicians, payers, and patients about the rigor of oversight for biosimilars will improve market uptake (Schneider et al., 2012). The positive experiences should strengthen reimbursement authorities, physicians, pharmacists, and patients to have a positive attitude toward biosimilars and trust in the EMA biosimilar pathway.

It is argued that a high discount strategy will not overcome the lack of physician confidence and might easily be countered by the originator company. The absence of demand-side policies for biosimilars in many EU member states restricts the potential price difference between originator and biosimilar (Simoens and Huys, 2013).

The inclusion of a biosimilar in treatment guidelines, e.g., filgrastim biosimilar in European Organization for Research and Treatment of Cancer (EORTC) guidelines (Aapro et al., 2016) and infliximab biosimilar in National Institute for Health and Care Excellence (NICE) guidance (NICE, 2016), will increase uptake.

The lack of incentive and burden of change barrier will become less high with the increase of experience and knowledge of biosimilars and the growing pressure on healthcare budgets.

#### Change attitude toward biosimilar switching/substitution, starting with physician, and patient education

Two possible ways are proposed to change the attitude toward biosimilar switching and substitution. First the realization and publication of more head-to-head studies with the reference product, and second strict regulations on biosimilar exchange quota (Haustein et al., 2012). The Norway Ministry of Health funded NOR-SWITCH to compare the biosimilar infliximab in all granted indications with its reference product Remicade® after switching from the reference product (Asbjørn, 2015). The rationale behind setting quota, that the prescription of biosimilar mAbs to a new patient or the one time switch for an existing patient may be considered as a low risk, will need to be clearly communicated, e.g., the position paper of the Finnish medicines agency (Fimea, 2015). This education is best done through pharmacists and physicians, who can function as ambassadors. This communication may cause an increased uptake apart from the subsequent quota introduction (Lepage-Nefkens et al., 2013). Information about biosimilars needs to be better spread to all concerned to raise confidence in the biosimilar development model. Members of the EMA Biosimilars Working Party have published two papers with a clear message that biosimilars can be considered therapeutic alternatives to the reference product (Weise et al., 2012, 2014).

However, it is unclear in view of the current knowledge whether back and forth switching may jeopardize patient safety. For now, it seems that introduction of biosimilars will be largely limited to new patients, or stable patients where a judiciously made one-time switch initiated by the prescriber can be made, making this barrier most relevant for those biosimilar mAbs with indications for long-term chronic use like rheumatoid arthritis.

#### Differentiate the biosimilar by service offerings, use an appropriate comparator in cost-effectiveness analyses

Biosimilars may be positioned as late-entrant branded products rather than generics. The biosimilar developers may need to develop strategies to differentiate products on the basis of branding and corporate identity by providing services linked to the brand identity (Ellery and Hansen, 2012). An optimized formulation (with e.g., better stability, less painful injections, more convenient storage conditions) and packaging variants and sizes are other ways a biosimilar developer can positively differentiate its biosimilar.

From a health-economic perspective, several aspects need to be considered. First, with a view to quantifying the value of second or third generations of biologics using the efficiency frontier approach (Cleemput et al., 2012), cost-effectiveness needs to be calculated versus the previous most cost-effective alternative. This alternative can be a first generation biologic or the biosimilar. Second, given that pharmaceutical companies (can) offer discounts/rebates on biologics and biosimilars, prices of biologics and biosimilars used in a cost-effectiveness analysis should be prices net of discounts/rebates. Third, economic pressure can result in lower incremental cost-effectiveness ratio (ICER) threshold limits (Cleemput et al., 2008). Therefore, second or third generation biologics will have to prove highly efficacious or cannot ask a premium price when a biosimilar or a first generation biologic is used as comparator in the cost-effectiveness analysis.

However, with the long development time and slow market uptake, the risk remains that an innovative treatment replaces the competitive power of a biosimilar mAb before the biosimilar has become profitable (Declerck, 2013).

Although brand manufacturers will still defend their reference products and compete with other biosimilar developers, they are now producing biosimilars as well. It is thus not in their interest to campaign indiscriminately against the concept of biosimilars. An example is how the acquisition of Hospira by Pfizer led to marketing of biosimilars via their Established Products Division, which is an independent competitive business unit. The innovator will still have a competitive advantage over other biosimilar developers, since biosimilar mAbs that are developed by innovator companies can benefit from the company's network and reputation.

The strong ties that innovator companies have with physicians through supporting investigator initiated trials can be challenged when the biosimilar developer has enough credibility, product and resources to facilitate these trials. This reach of innovator barrier will become less relevant once biosimilar mAbs are more accepted as therapeutic alternatives, but will never cease to exist.

## Conclusion

Our literature search found evidence that market access of biosimilar mAbs in the EU is hampered by six barriers: The manufacturing process, the regulatory process, intellectual property rights, lack of incentive, the impossibility of substitution and the innovator's reach. (Table 2)

All of the barriers mentioned above apply more or less to all biosimilars, which have accordingly seen slow uptake in European markets (GaBI, 2012; Farfan-Portet et al., 2014).

Based on the literature search the following recommendations can be done to overcome these barriers to the market access of biosimilar mAbs (Table 3):

1. Invest initially in advanced production processes with the help of single-use technology, buy-in of experience or outsourcing.
2. Gain experience with the regulatory process and establish alignment between stakeholders.
3. Limit patent litigation, eliminate evergreening benefits, build out further the unitary patent and unified patent litigation system within the EU.
4. Create demand-side policies, disseminate objective information.
5. Change attitude toward biosimilar switching/substitution, starting with physician and patient education.
6. Differentiate the biosimilar by service offerings, use an appropriate comparator in cost-effectiveness analyses.

In addition to the recommendations above, the authors believe that within the group of biosimilar mAbs, products of which the mode of action is better understood, and/or of which the risk of immunogenicity is thought to be less, the competition is high, and which are used in short-term treatments, are expected to be accepted by the market more easily. They are thus expected to experience faster uptake than products for which the opposite is valid.

Since biosimilars are approved on a European level, it would be a good idea to make recommendations on interchangeability on a European level as well, to support national authorities in policy development and decision making.

Future research could investigate market dynamics, since the market is evolving rapidly. Not only the relationship innovator/biosimilar can be studied, also differences with second-generation innovator products. Barriers to the market access of biosimilar mAbs could be reduced when more transparency and communication/education is used in all steps toward market access in order to increase the trust in biosimilar mAbs by all relevant stakeholders. Only then biosimilar mAbs will be able to fully live up to their cost saving potential.

## Author contributions

EM and CJ reviewed the literature and drafted the initial version of the manuscript. SS, IH, PD, and AV revised the manuscript critically and contributed to the interpretation of the identified barriers and recommendations to overcome these. All authors read and approved the final manuscript.

### Conflict of interest statement

SS, IH, PD and AV are the founders of the KU Leuven Fund on Market Analysis of Biologics and Biosimilars following Loss of Exclusivity. SS, IH and AV are conducting biosimilar research sponsored by Hospira. SS, IH and EM are involved in a stakeholder roundtable on biosimilars sponsored by Amgen and MSD. SS has participated in an advisory board meeting on biosimilars for Pfizer. AV is involved in consulting, advisory work and speaking engagements for a number of companies, a.o. AbbVie, Amgen, Biogen, EGA, Pfizer/Hospira, Mundipharma, Roche, Sandoz. He has no personal benefit from these activities; any compensation is paid to his employer. PD participated at advisory board meetings for AbbVie, Amgen, and Hospira and is on the Speakers' Bureau of AbbVie, Celltrion, Hospira, Merck Serono, and Roche. CJ declare that the research was conducted in the absence of any commercial or financial relationships that could be construed as a potential conflict of interest.
